# Ginsenoside Re Inhibits ROS/ASK-1 Dependent Mitochondrial Apoptosis Pathway and Activation of Nrf2-Antioxidant Response in Beta-Amyloid-Challenged SH-SY5Y Cells

**DOI:** 10.3390/molecules24152687

**Published:** 2019-07-24

**Authors:** Meichen Liu, Xueyuan Bai, Shiting Yu, Wenxue Zhao, Juhui Qiao, Ying Liu, Daqing Zhao, Jiawen Wang, Siming Wang

**Affiliations:** Jilin Ginseng Academy, Changchun University of Chinese Medicine, Changchun 130117, China

**Keywords:** Alzheimer’s disease, β-amyloid, ginsenoside Re, mitochondria, oxidative stress

## Abstract

Accumulation of amyloid-β (Aβ), which results in the formation of senile plaques that cause oxidative damage and neuronal cell death, has been accepted as the major pathological mechanism of Alzheimer’s disease (AD). Hence, inhibition of Aβ-induced oxidative damage and neuronal cell apoptosis represents the effective strategies in combating AD. Ginsenoside Re (Re) has pharmacological effects against Aβ-induced neurotoxicity. However, its molecular mechanism remains elusive. The present study evaluated the effect of Re against Aβ-induced cytotoxicity and apoptosis in SH-SY5Y cells, and investigated the underlying mechanism. We demonstrate that Re inhibits the Aβ-triggered mitochondrial apoptotic pathway, as indicated by maintenance of mitochondrial functional, elevated Bcl-2/Bax ratio, reduced cytochrome c release, and inactivation of caspase-3/9. Re attenuated Aβ-evoked reactive oxygen species (ROS) production, apoptosis signal-regulating kinase 1 (ASK1) phosphorylation, and JNK activation. ROS-scavenging abrogated the ability of Re to alter ASK-1 activation. Simultaneously, inhibition of JNK abolished Re-induced Bax downregulation in Aβ-challenged SH-SY5Y cells. In addition, Re enhanced activation of the nuclear factor-E2-related factor 2 (Nrf2) in Aβ-induced SH-SY5Y cells. Knockdown of Nrf2 by small interfering RNA targeting Nrf2 abolished the protective effect of Re. Our findings indicate that Re could be a potential therapeutic approach for the treatment of AD.

## 1. Introduction

Alzheimer’s disease (AD), which affects millions of older adults worldwide, is the most prevalent chronic neurodegenerative disorder [1]. The number of patients with AD is rapidly increasing such that by 2050, the number of individuals with AD will exceed 130 million worldwide [2]. Increased β-amyloid (Aβ) plaques in the brain are regarded as the pathological hallmark of AD [3]. The self-assembling of Aβ into neurotoxic aggregates is considered a central event in the pathogenesis of AD (amyloid hypothesis). Several adverse factors are known to contribute to Aβ aggregation. Current research indicates that abnormal interactions with model membranes have been evidenced to foster Aβ aggregation [4]. Mislocated metal ions as Cu(II), which in physiological conditions contribute to the stability of native proteins, may accelerate reactive oxygen species (ROS) production, protein misfolding, and aggregation [5]. Although the pathogenesis of AD remains largely unclear, the amyloid hypothesis remains dominant. However, there is no treatment available that substantially delays the onset or progression of AD [6,7,8]. Thus, treating AD is the single biggest unmet medical need in neurology and the development of an effective therapeutic intervention is crucial for public health.

Apoptosis is a physiological response that occurs during the development of the nervous system [9]. Aberrant apoptosis plays a key role in the progression of several neurodegenerative disorders [10]. Autopsy of AD patients revealed large amounts of neuronal apoptosis in the brain, and Aβ-induced neuronal apoptosis has been recapitulated in various models of AD [11,12,13]. Notably, mitochondria are at the center stage in human neurodegenerative diseases. Accumulating evidence has implicated mitochondria in Aβ-induced neuronal apoptosis. Indeed, defects in mitochondria trigger oxidative stress, which is the primary event in AD pathology [14]. Defective mitochondria inhibit the production of ATP and increase the production of ROS [15]. Accumulation of ROS leads to oxidative damage, which accelerates Aβ accumulation, activates the mitochondrial permeability transition pore, increases cytochrome c (cyt c) release, and subsequently induces mitochondria-related apoptotic pathways [16]. Apoptosis signal-regulating kinase 1 (ASK-1), a key regulator of the mitochondria-related apoptotic pathway, is highly sensitive to ROS and plays a pivotal role in neuroprotection by regulating the Bcl-2 family of proteins [17,18]. Apart from this, the neuronal membranes are found to be rich in polyunsaturated fatty acids, which are highly susceptible to ROS. Accumulation of ROS result in biochemical alteration in bimolecular components, further linked to a variety of potential toxic mechanism associated with AD [19,20,21]. Although it remains unclear whether oxidative stress is a major cause or merely a consequence of mitochondrial dysfunction associated with AD, supplementation with antioxidants is reportedly beneficial, especially in the early stages of AD [22,23]. Thus, pharmacological inhibition of oxidative stress and regulation of mitochondria-related apoptotic pathways in AD pathology have sparked interest as therapeutic targets.

To this aim, several natural antioxidants as silymarin, bacoside-A, and resveratrol have evidenced antioxidant effects and are currently under clinical investigation [24,25,26,27]. Among natural antioxidants, *Panax ginseng* C.A. Meyer (*P. ginseng*) is an herb that has been used in China for thousands of years. *P. ginseng* has excellent antioxidant effects, as well as demonstrated pharmacological effects in the central nervous system [28,29]. Ginsenosides are the major active components responsible for the multiple activities of ginseng [30]. Ginsenoside Re, one of the most important active components of ginsenosides, possesses antioxidant and antioxidant-related properties in various cell types [31,32,33]. Previous studies reported that ginsenoside Re exhibited direct neuroprotective effects against Aβ stimulation. Indeed, ginsenoside Re significantly reduced Aβ1–40 and Aβ1–42 levels in cell-based assays, and its oral administration significantly reduced Aβ levels in the brains of Tg2576 mice [34]. Thus, ginsenoside Re may provide a potential means of slowing the progression of AD, although the underlying cellular and molecular mechanisms are still unclear. We speculated that the neuroprotective activity of ginsenoside Re might be attributable to its dual antioxidant and anti-apoptotic activities. To address this hypothesis, we characterized the neuroprotective activities of ginsenoside Re in Aβ-induced neurotoxicity in SH-SY5Y cells, and investigated involved signaling pathways. We first demonstrated that the neuroprotective activity of ginsenoside Re was closely associated with the regulation of ROS-dependent ASK-1/c-Jun N-terminal kinases (JNK)/Bax apoptosis pathways. We also demonstrated that ginsenoside Re activated the endogenous antioxidant response pathways by activating Nrf2 and its target genes against Aβ-induced oxidative stress.

## 2. Results

### 2.1. Ginsenoside Re Protected SH-SY5Y Cells against Aβ25–35-Induced Cytotoxicity

**Figure 1:**
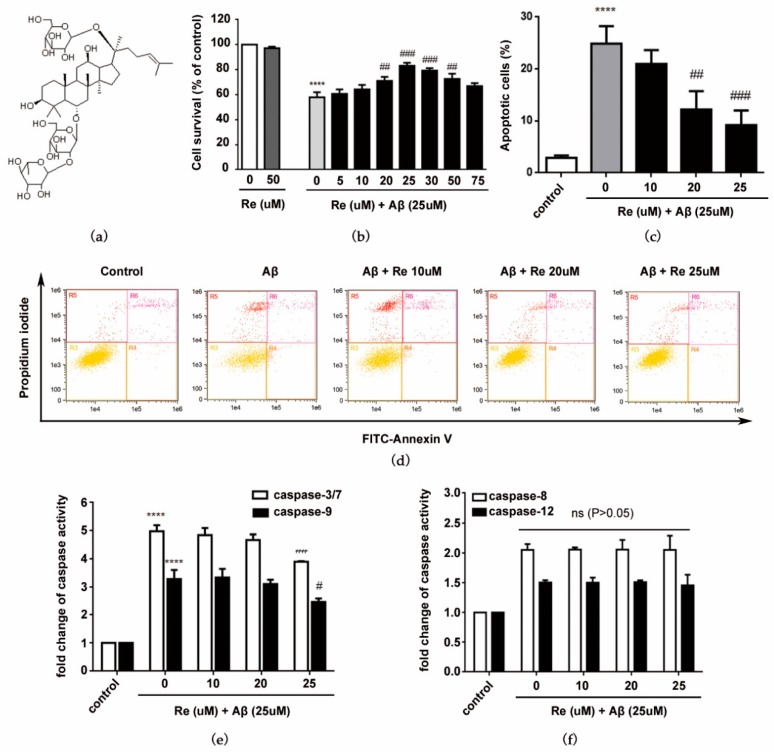
Protective effects of ginsenoside Re against Aβ25–35-induced cell damage. (**a**) Chemical structure of ginsenoside Re (molecular formula: C48H82O18; molecular weight: 947.12); (**b**) SH-SY5Y cells were co-treated with ginsenoside Re and Aβ for 24 h. Cell viability was determined by CCK-8 assays. Bar diagrams showing percentage of survival cells; (**c**) bar diagrams showing percentage of apoptosis; (**d**) annexin V/FITC-PI staining and flow cytometric analysis of apoptosis; (**e**) caspase-3/7 and caspase-9 activity were assessed by fluorometric and colorimetric assay, respectively; and (**f**) caspase-8 and caspase-12 activity were assessed by fluorometric assay. **** *p* < 0.0001 versus control; ^#^ *p* < 0.05, ^##^ *p* < 0.01, ^###^ *p* < 0.001 versus Aβ treatment alone.

Ginsenoside Re reportedly shows neuroprotective effects in vitro and in vivo [34]. Thus, we used the CCK-8 method to quantify cytoprotection elicited by ginsenoside Re. As shown in Figure 1b, without Aβ25–35 treatment, ginsenoside Re (75 uM) showed no toxicity to SH-SY5Y cells. When treated with 25 uM Aβ25–35, the cell viability significantly decreased. In the range (20, 25, and 30 uM), ginsenoside Re has a promotive effect on neuronal cell survival. 25 µM ginsenoside Re reached a peak with a 43.51% increase compared with Aβ-induced alone. Therefore, 25 μM was chosen as the maximum concentration of ginsenoside Re for use throughout subsequent experiments.

As Aβ-induced apoptosis is a key pathologic event in AD [12], we next investigated whether ginsenoside Re inhibited Aβ-induced apoptosis of SH-SY5Y cells. Results of Annexin V-FITC/PI staining and flow cytometry revealed that apoptotic rates were decreased from 25.88% to 10.23% compared with Aβ-induced SH-SY5Y cells (Figure 1c,d). The effect of ginsenoside Re on apoptotic markers was further examined. Ginsenoside Re significantly reduced Aβ-induced increases in caspase-3/7 activity and decreases in caspase-9 activity, but did not significantly affect caspase-8 or caspase-12 activities (Figure 1e,f). These results suggest that the mitochondrial pathway (intrinsic), rather than the endoplasmic reticulum or death receptor pathway (extrinsic), contributes to the neuroprotective effect of ginsenoside Re.

### 2.2. Ginsenoside Re Alleviated Mitochondrial Dysfunction and Prevented the Mitochondria-Mediated Apoptotic Pathway in SH-SY5Y Cells after Aβ25–35 Exposure

We demonstrated that ginsenoside Re protected SH-SY5Y cells against Aβ-induced apoptosis through the mitochondrial pathway. Thus, we next investigated the role of ginsenoside Re in mitochondrial dysfunction and downstream mitochondria-mediated apoptosis-related proteins in response to Aβ exposure.

**Figure 2:**
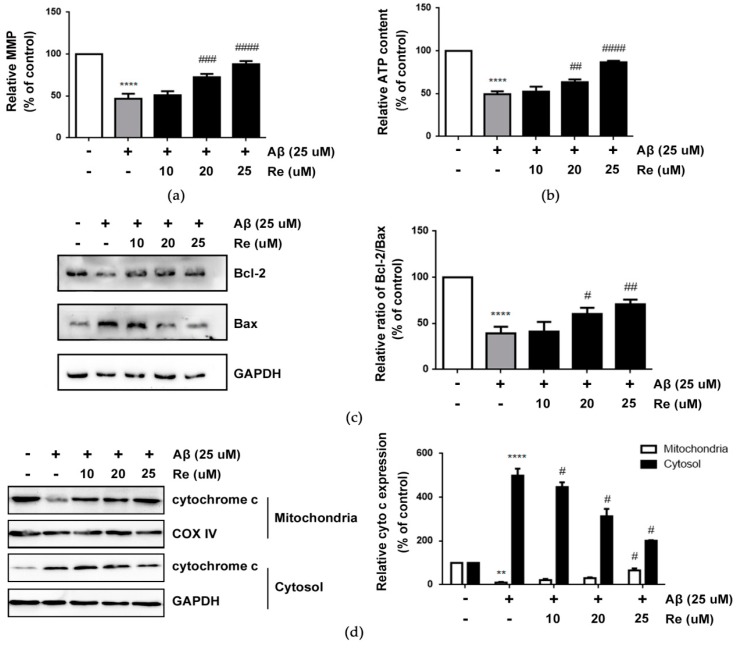
Ginsenoside Re alleviated mitochondrial dysfunction and prevents mitochondria mediated apoptotic pathway after Aβ 25–35 exposure in SH-SY5Y cells. (**a**) The loss of MMP in SH-SY5Y cells; (**b**) the relative ATP content in SH-SY5Y cells; (**c**) the protein expression levels of Bcl-2 and Bax in SH-SY5Y cells were detected by western blot analysis; (**d**) western blot analysis to detect cytochrome c levels in mitochondrial fraction and cytosolic fraction of SH-SY5Y cells. ** *p* < 0.01, **** *p* < 0.0001 versus control; ^#^ *p* < 0.05, ^##^ *p* < 0.01, ^###^ *p* < 0.001, ^####^ *p* < 0.0001 versus Aβ treatment alone.

Collapse of MMP (mitochondrial membrane potential) is an early step in the induction of mitochondrial dysfunction that can subsequently induce apoptosis [35]. Our results demonstrated that Aβ-exposure resulted in the loss of MMP, but ginsenoside Re prevented this effect in Aβ-treated SH-SY5Y cells (Figure 2a). In addition, ginsenoside Re rescued the reduction of ATP production in Aβ-treated SH-SY5Y cells (Figure 2b).

As Bax coordinates with Bcl-2 to trigger the mitochondrial apoptotic pathway [36], we next examined whether ginsenoside Re altered expression of Bcl-2 or Bax. The results showed that ginsenoside Re pre-treatment increased the ratio of Bcl-2/Bax compared with Aβ-induced SH-SY5Y cells (Figure 2c). As Bcl-2/Bax induces release of cyt c from mitochondria into the cytoplasm when mitochondrial apoptosis occurs, we analyzed cyt c release by western blot analysis [37]. Our results showed increased cytosolic cyt c levels increased in Aβ25–35-induced SH-SY5Y cells. Consistent with expectations, ginsenoside Re pre-treatment prevented cyt c release from mitochondria into the cytosol (Figure 2d). These results demonstrated that the neuroprotective effect of ginsenoside Re is accompanied by the protection of mitochondrial dysfunction and prevention of the mitochondria-mediated apoptotic pathway.

### 2.3. Ginsenoside Re Inhibited Aβ-induced ASK-1/JNK Activation in a ROS-Dependent Manner

**Figure 3:**
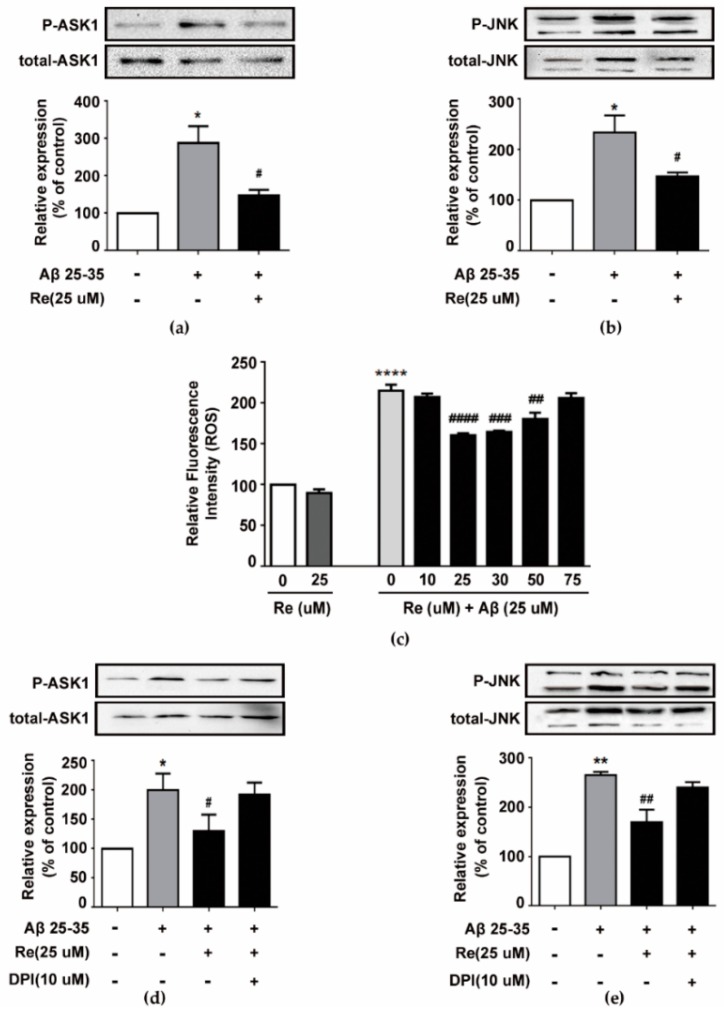
Inhibition of Aβ-induced ASK1/JNK pathway by ginsenoside Re is dependent on attenuation of Aβ-induced excessive reactive oxygen species (ROS) generation. (**a**) Level of p-ASK1 was analyzed by western blot and the band intensity of p-ASK1 was quantified by densitometry and normalized to total ASK1; (**b**) level of p-JNK was analyzed by western blot and the band intensity of p-JNK was quantified by densitometry and normalized to total JNK; (**c**) intracellular ROS were estimated using the fluorescent probe DCFH-DA by Amnis flow cytometry; (**d**,**e**) SH-SY5Y cells were pre-treated with or without DPI (NADPH oxidase inhibitor diphenyleneiodonium) for 50 min and then treated with Re in the presence or absence of Aβ for 24 h. Level of p-JNK and p-ASK1 was analyzed by western blot. * *p* < 0.05, ** *p* < 0.01, **** *p* < 0.0001 versus control; ^#^ *p* < 0.05, ^##^ *p* < 0.01, ^###^ *p* < 0.001, ^####^ *p* < 0.0001 versus Aβ treatment alone.

Ginsenoside Re dramatically attenuated Aβ-induced Bax upregulation in a concentration-dependent manner; thus, we further investigated its upstream mechanism. Bax expression is regulated by the ASK-1/JNK pathway, whose activation contributes to Aβ-induced apoptosis [38,39]. Thus, we hypothesized that the ASK-1/JNK pathway is involved in the neuroprotective effects of ginsenoside Re. We examined phosphorylated and total protein levels of ASK-1 and JNK in Aβ-induced SH-SY5Y cells treated with ginsenoside Re by western blot assay. As shown in Figure 3a,b, 25 µM ginsenoside Re obviously attenuated Aβ-induced phosphorylation of ASK-1 and JNK.

Previous reports suggested that Aβ-induced ASK-1 activation and apoptosis is mediated by ROS [40]. Moreover, excessive ROS is generated in Aβ-challenged cells. Therefore, we examined whether ginsenoside Re modulated ROS production in Aβ-challenged SH-SY5Y cells. Our data showed that cells exposed to Aβ for 24 h exhibited an approximately two-fold increase in intracellular ROS, but ginsenoside Re treatment prevented ROS elevation in a dose-dependent manner (Figure 3c). Moreover, ginsenoside Re inhibited ASK-1/JNK phosphorylation in a ROS-dependent manner (Figure 3d,e).

### 2.4. Inhibition of ROS-Dependent ASK-1/JNK Signaling Attenuated the Neuroprotective Effects of Ginsenoside Re

**Figure 4:**
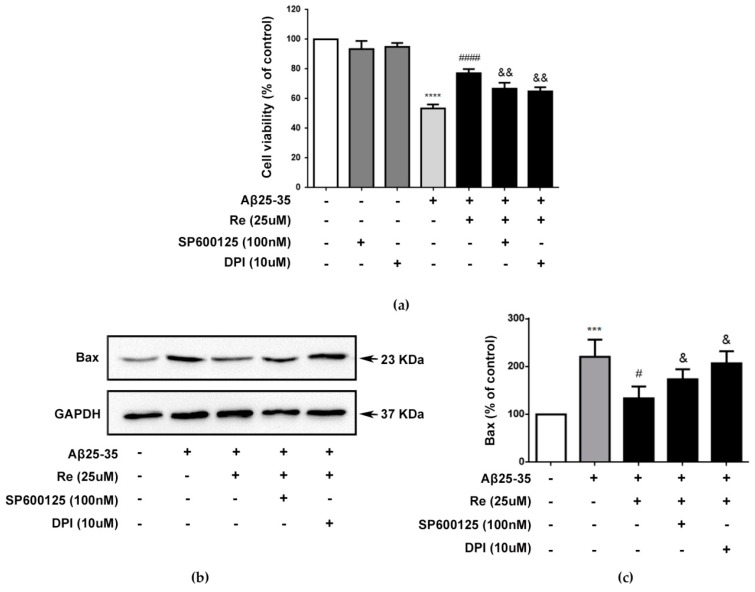
The neuroprotective effect of ginsenoside Re is dependent of ROS-dependent ASK1/JNK signaling pathway. (**a**) SH-SY5Y cells were pre-incubated with or without 100 nM sp600125 or 10 uM DPI for 50 min and then treated with 25 µM Re and with or without Aβ25–35 for 24 h. Cell viability was detected by CCK-8 assay; (**b**,**c**) western blot analysis to detect level of Bax. The band intensity of Bax was quantified by densitometry and normalized to GAPDH. *** *p* < 0.001, **** *p* < 0.0001 versus control; ^#^ *p* < 0.05, ^####^ *p* < 0.0001 versus Aβ treatment alone; ^&^ *p* < 0.05, ^&&^ *p* < 0.01 versus the cells treated with Aβ and 25 uM Ginsenoside Re.

To further confirm the roles of ROS-dependent ASK-1/JNK signaling pathway in the neuroprotective effect of ginsenoside Re, we tested whether pathway inhibitors could affect the cytoprotective effect of ginsenoside Re in Aβ-challenged SH-SY5Y cells. Our results revealed that 25 µM Re yielded about 30.6% neuroprotection compared with Aβ treatment alone, similar to the result shown in Figure 1a. However, addition of DPI (NADPH oxidase inhibitor diphenyleneiodonium) or sp600125 abolished the neuroprotective effect of Re (Figure 4a).

We next investigated whether these pathway inhibitors could affect Bax protein levels. Our results showed that ginsenoside Re-mediated inhibition of Bax expression in Aβ-challenged SH-SY5Y cells was dependent on ROS-dependent ASK-1/JNK signaling (Figure 4b,c).

### 2.5. Ginsenoside Re Attenuated Aβ-Induced Cellular Oxidative Stress by Activating Nrf2-Antioxidant Signaling

Ginsenoside Re reduced ROS levels and, thus, protected SH-SY5Y cells against Aβ-induced cytotoxicity. As such, we further investigated its antioxidative mechanisms. Nrf2 is recognized to regulate the expression of antioxidant genes that protect against oxidative damage triggered by injury. Thus, we next investigated involvement of the Nrf2 pathway in the antioxidative activity of ginsenoside Re.

**Figure 5:**
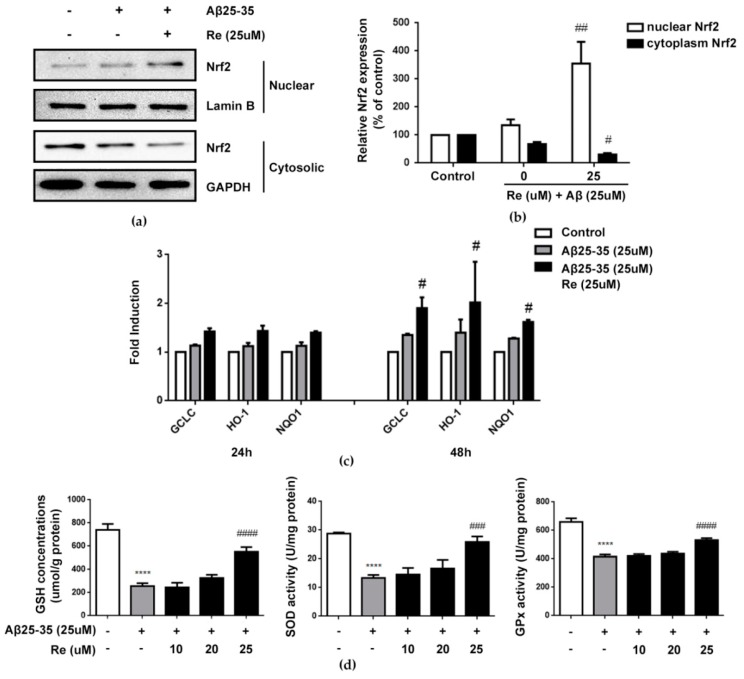
Ginsenoside Re induced the activation of Nrf2-antioxidant signaling in Aβ-induced SH-SY5Y cells. (**a**,**b**) Ginsenoside Re promoted the nuclear translocation of Nrf2 in Aβ-induced SH-SY5Y cells by western blot assay; (**c**) Aβ treatment significantly induced the expression of Nrf2-mediated antioxidant response genes, including *GCLc*, *NQO1* and *HO-1* at both 24 h and 48 h. Ginsenoside Re treatment potentiated this increase at both time points than Aβ induced alone (*n* = 3 per treatment condition); (**d**) the concentrations of GSH and activities of SOD and GPx in SH-SY5Y cells in different treatments were measured as described in the materials and methods (*n* = 5 per treatment condition). **** *p* < 0.0001 versus control; ^#^ *p* < 0.05, ^##^ *p* < 0.01, ^###^ *p* < 0.001, ^####^ *p* < 0.0001 versus Aβ treatment alone.

Our results revealed that 25 µM ginsenoside Re treatment markedly increased the level of Nrf2 protein in the nucleus and elicited a concomitant decrease in the cytoplasm (*p* < 0.01, *p* < 0.05, Figure 5a,b). Translocation of activated Nrf2 from the cytosol into nuclei induced the transcription of many Nrf2-regulated antioxidant enzymes. The effects of ginsenoside Re on mRNA expression of GCLc, HO-1, and NQO1 were determined, as they are directly linked to the Nrf2-mediated antioxidative defense response. *GCLc*, *HO-1* and *NQO1* gene expression was induced 24 h after Aβ administration and remained increased at 48 h post-treatment. Ginsenoside Re treatment further increased this expression. The effect of ginsenoside Re was even greater at 48 h than observed at 24 h in the presence of Aβ (*p* < 0.05 versus cells treated with Aβ25–35 alone; Figure 5c). Consistently, the content of GSH and activities of SOD and Gpx were significantly increased by ginsenoside Re treatment (*p* < 0.0001 versus control, *p* < 0.001, *p* < 0.0001 versus cells treated with Aβ25–35 alone Figure 5d).

### 2.6. Ginsenoside Re Alleviated Aβ-Induced Cytotoxicity and ROS Generation in SH-SY5Y Cells in a Nrf2-Dependent Manner

**Figure 6:**
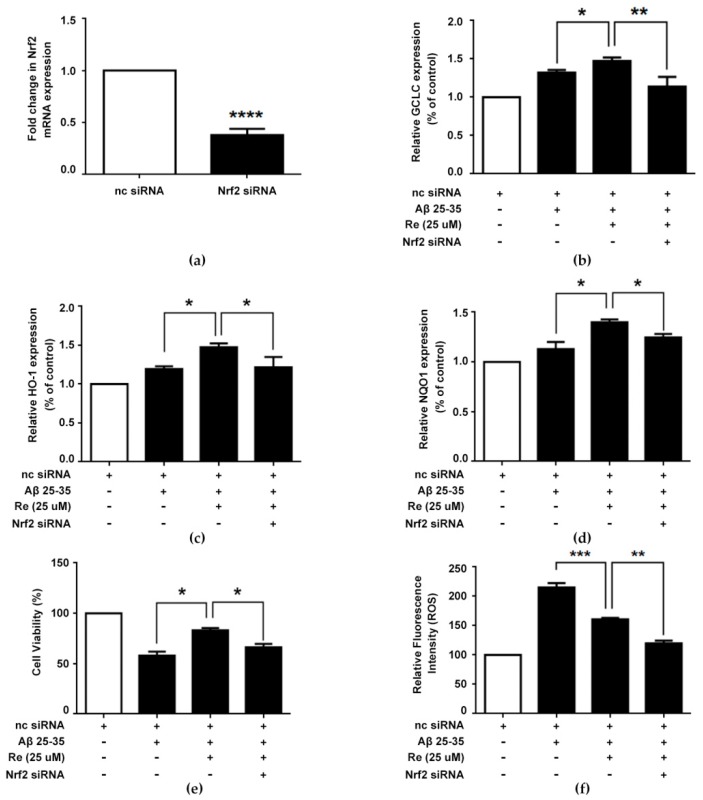
Ginsenoside Re alleviated Aβ-induced cytotoxicity and ROS generation in SH-SY5Y cells through Nrf2-dependent manner. (**a**) *Nrf2* gene expression in negative control (nc) siRNA treated- and NRF2 siRNA treated group; (**b**–**d**) The relative mRNA expression of antioxidative enzymes were measured by qRT-PCR; (**e**) after transient transfection with or without Nrf2 siRNA plasmid, SH-SY5Y cells were treated with ginsenoside Re in the presence or absence of Aβ for 24 h. Cell viability was detected by CCK-8 assay; (**f**) intracellular ROS were estimated using the fluorescent probe DCFH-DA by flow cytometry. * *p* < 0.05, ** *p* < 0.01, *** *p* < 0.001, **** *p* < 0.0001 versus control.

Based on the outcomes described above, we hypothesized that the protective effects of ginsenoside Re against Aβ-induced oxidative damage resulted from upregulation of the Nrf2 pathway. To verify this hypothesis, Nrf2 was silenced in Aβ-induced SH-SY5Y cells (Figure 6a). Quantification of antioxidative enzyme expression suggested that the effects of ginsenoside Re on antioxidative enzyme expression were almost abolished in Nrf2-silenced cells (Figure 6b–d).

Additionally, we determined the effect of ginsenoside Re on Aβ-induced cell viability and ROS generation in Nrf2-silenced cells. Our results indicated that the cytoprotective effect on cell viability and inhibitory effect on ROS generation of ginsenoside Re were substantially impeded by Nrf2 silencing (Figure 6e,f). Collectively, these results demonstrated that ginsenoside Re-mediated cytoprotection might also be associated with targeting of the Nrf2 pathway.

## 3. Discussion

Despite significant efforts to discover a therapeutic strategy to prevent the progression of or cure AD, available therapy is limited to symptomatic treatments whose efficacy remains unsatisfactory at present. The results of the present study demonstrate, for the first time, that ginsenoside Re prevents Aβ-induced mitochondrial-dependent apoptosis in SH-SY5Y cells by increasing their cellular Bcl-2/Bax ratio. The upstream mechanism was associated with attenuation of ROS-dependent proapoptotic Bax upregulation that occurs under apoptotic challenge: Ginsenoside Re inhibited Bax expression in Aβ-challenged SH-SY5Y cells by inhibiting JNK activity via ASK-1 inactivity. Further investigation revealed that this protective effect of ginsenoside Re was associated with activation of Nrf2-antioxidant signaling. Indeed, ginsenoside Re could induce nuclear translocation of Nrf2 to enhance the activities of several antioxidant enzymes.

Natural products, which tend to have fewer side effects, have been an important source for the discovery of novel therapeutically active compounds for AD [41]. *P. ginseng*, a primary herb in Traditional Chinese Medicine, has been used to treat a wide array of diseases—especially neurodegenerative diseases [42]. Ginsenosides are the most important active ingredients in *P. ginseng* and previous studies have shown their beneficial effects on AD [43]. Ginsenoside Re, a highly abundant active component of ginsenosides, has been found to have neuroprotective effects in a variety of animal models, including AD. Ginsenoside Re attenuated β-amyloid and serum-free-induced neurotoxicity in PC12 cells and decreased levels of Aβ1–40 and Aβl-42 in the brains of Tg2576 mice [34,44]. In the present manuscript, we extended these observations by exploring the mechanism of Aβ-induced neuroprotection. Identifying the molecular targets of ginsenoside Re would be useful for anti-AD drug development.

It has been accepted that Aβ accumulation causes mitochondria dysfunction that induces oxidative stress and excessive apoptosis, thus forming the primary event in AD pathology. As such, targeting the Aβ-induced mitochondria-mediated apoptotic pathway and oxidative stress may be effective therapeutic strategies to attenuate Aβ-induced neurotoxicity and, thus, improve neurological outcomes in AD. Aβ1–42 is the major peptide constituent of amyloid plaques. However, Aβ25–35 is the shortest fragment that exhibits large β-sheet fibrils and retains the toxicity of the full-length peptide. Therefore, Aβ25–35 is widely used instead of the endogenous Aβ1–42 fragment in AD-relevant insults in vitro [45]. In this study, Aβ25–35 has been chosen as a model for full-length Aβ because it retains both its physical and biological properties, while its short length readily allows derivatives to be studied. Consistent with previous reports, we observed Aβ25–35 toxicity in SH-SY5Y cells. Co-treatment with ginsenoside Re rescued effects on cell viability and excessive apoptosis. Apoptotic cell death involves the activation of various caspases, which play a main role in activating and regulating the whole apoptosis process. Ginsenoside Re significantly blocked Aβ-induced increases in caspase-3 and caspase-9 (mitochondria-associated caspase) activity, but failed to affect the activity of caspase-8 (a death receptor and apoptotic signaling-related caspase) or caspase-12 (an endoplasmic reticulum-specific stress-activated caspase). These results suggest that the mitochondrial pathway plays a major role in the neuroprotective properties of ginsenoside Re against Aβ-induced toxicity in SH-SY5Y cells.

This function of mitochondria, which are direct targets for Aβ, is often disrupted in an early phase of AD [46]. Mitochondrial dysfunction is consistent with the reduction of MMP, which induces opening of the mitochondrial permeability transition pore, further causing release of cyt c into the cytosol. The results of our study demonstrated that ginsenoside Re prevented the loss of MMP, suppressed cyt c release, and promoted ATP production, indicating that ginsenoside Re protected SH-SY5Y cells against Aβ25–35-induced injury through inhibition of mitochondrial dysfunction. Apoptosis is preceded by changes in regulatory proteins, such as Bcl-2 (anti-apoptotic) and Bax (pro-apoptotic), which are located in the mitochondrial membrane. The ratio of Bcl-2 to Bax is a critical factor for mitochondria-mediated apoptosis. The current study found that ginsenoside Re could significantly elevate the Bcl-2/Bax ratio in Aβ-challenged cells.

What is the signaling mechanism by which ginsenoside Re increases the Bcl-2/Bax ratio under apoptotic challenge? Bax is a downstream gene of ASK-1. Under normal conditions, ASK-1 is kept in an inactive form by reduced thioredoxin (Trx), which regulates ASK-1 activation in a redox-sensitive manner. Trx becomes oxidized and separates from ASK-1 when oxidative stress occurs and intracellular ROS levels increase. Subsequently, ASK-1 forms a homo-oligomeric complex, which leads to full activation of ASK-1 through autophosphorylation. Activated ASK-1 further activates JNK and upregulates Bax expression [47]. In this study, ASK-1 and JNK were significantly activated in Aβ25–35-treated SH-SY5Y cells, which were probably triggered by increased intracellular ROS levels. However, activation of ASK-1 and JNK were inhibited by ginsenoside Re treatment, which is consistent with the results of ROS detection. Use of the pharmacological inhibitor DPI revealed that ginsenoside Re suppressed ASK-1 and JNK activation through a ROS-dependent mechanism. Importantly, the inhibition of apoptosis elicited by ginsenoside Re could partly be blocked by either DPI of the JNK inhibitor SP600125, indicating that ginsenoside Re can inhibit Aβ25–35-induced apoptosis-promoting signals, partly by neutralizing Aβ-induced oxidative stress.

How does ginsenoside Re inhibit ROS generation to prevent the apoptotic effects of Aβ? Nrf2 is a stress-responsive transcriptional factor and key effector that protects cells from oxidative injury, particularly in neurodegenerative diseases [48]. Under oxidative stress conditions, Nrf2 translocates from the cytoplasm into the nucleus, whereby it subsequently induces the expression of a series of proteins, including phase II detoxifying enzymes and antioxidant proteins that can further enhance the anti-oxidative capabilities of cells [49]. Here, we demonstrated that ginsenoside Re remarkably promoted Nrf2 nuclear translocation and upregulated a set of genes encoding phase-II enzymes including HO-1, NQO1, and GCLc-consistent with increased concentrations of GSH and activities of SOD and GPx. The role of Nrf2 in Aβ-induced ROS is controversial. Ramsey and colleagues reported that Nrf2 is primarily located in the cytoplasm and reduced in nuclei of AD brains, suggesting that Nrf2 does not actively induce the expression of antioxidant enzymes in AD brains [50]. Sarkar and colleagues demonstrated that Aβ25–35 produced significant oxidative stress and downregulated Nrf2 levels [51]. In our study, exposure to Aβ25–35 caused a marginal increase in Nrf2 activation. This discrepancy might result from differences in dose and time differences, as well as the procedure used for Aβ exposure. Furthermore, specific knockdown of Nrf2 using Nrf2-siRNA abolished ginsenoside Re-induced upregulation of antioxidant enzymes and almost completely disabled the neuroprotective effects of ginsenoside Re, suggesting that its anti-oxidative and neuroprotective effects are related to activation of the Nrf2 signaling pathway.

**Figure 7:**
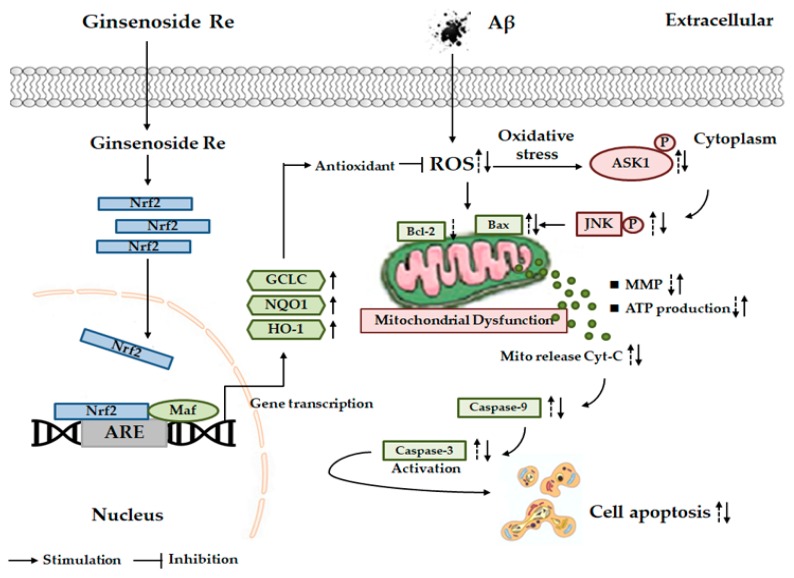
Scheme summarizing the protection of Aβ25–35-induced SH-SY5Y cell mitochondrial dysfunction by ginsenoside Re via inhibits ROS/ASK-1 dependent mitochondrial apoptosis pathway and activation of Nrf2-antioxidant response pathway.

In summary, we demonstrated that ginsenoside Re exhibited strong neuroprotective activity against Aβ25–35-induced neurotoxicity in SH-SY5Y cells by inhibiting ROS-dependent ASK-1/JNK/BAX cell apoptosis and activating Nrf2/HO-1 antioxidant pathways (Figure 7). These data support a potential role for ginsenoside Re in the prevention and treatment of AD. Nevertheless, the underlying mechanisms are certainly more complex than described here. For example, our results do not exclude other potential mechanisms involved in ginsenoside Re activation of Nrf2. Moreover, while we acknowledge that in vitro models may not be clinically relevant, the present results suggest that ginsenoside Re may be a promising candidate for the treatment of AD.

## 4. Materials and Methods

### 4.1. Drug Preparation

Ginsenoside Re (purity ≥98%) was purchased from the Chengdu Must Bio-Technology Co., Ltd. (Sichuan, China). The molecular structure of ginsenoside Re is shown in Figure 1a.

Aβ25–35 (Aβ) (Sigma, St. Louis, MO, USA) was diluted to 1 mM in dimethylsulfoxide, incubated with constant oscillation for seven days at 37 °C to induce aggregation, and then stored at −20 °C until use. To prepare for use, it was further diluted to 25 μM in culture medium.

### 4.2. Cell Culture

SH-SY5Y human neuroblastoma cells (purchased from ATCC (Rockville, MD, USA)) were cultured in DMEM/F12 media supplemented with 10% fetal bovine serum and 1% penicillin-streptomycin at 37 °C with 5% CO_2_. The medium was changed every other day, and cells were plated at an appropriate density according to each experimental scale.

### 4.3. Analysis of Cell Viability

Cell viability was analyzed with a CCK-8 kit (Dojindo Laboratories, Kumamoto, Japan) according to the manufacturer’s instructions. SH-SY5Y cells were seeded at 0.2 × 105 cells/well in 96-well plates. After 24 h of incubation to allow cells to adhere, cells were co-treated with ginsenoside Re (0, 5, 10, 20, 25, 30, 50, or 75 µM) and 25 µM Aβ for 24 h. After incubation, 20 μL of CCK-8 solution was added to cells and incubated for 1 h at 37 °C. Absorbance in each well was quantified at 450 nm using a microplate reader (Tecan, Salzburg, Austria).

### 4.4. Inhibitor Treatment and siRNA Transfection

In experiments involving the inhibition of JNK or ROS, sp600125 (100 nM) or DPI (10 µM) was added 50 min prior to ginsenoside Re and/or Aβ25–35 treatment. Cells were transfected with control siRNA or Nrf2 siRNA using a customized siRNA reagent system (RiboBio, Guangzhou, China) according to the manufacturer’s instructions.

### 4.5. Cell Apoptosis Analysis

Cell apoptosis was measured using an Annexin-V-FITC Apoptosis Detection Kit (BD Biosciences, San Jose, CA, USA). SH-SY5Y cells were treated as described above, harvested with 0.25% trypsin, washed twice with phosphate-buffered saline (PBS), resuspended in buffer, and incubated with 5 μL Annexin V-FITC and 5 μL propidium iodide (PI) for 15 min in the dark at room temperature. Samples were then analyzed by flow cytometry (Amnis, Seattle, WA, USA).

### 4.6. Caspase Activity Assay

Caspase activity was assayed using a Caspase-3 and Caspase-8 Assays (Promega, Madison, WI, USA), a Caspase-9 Activity Assay Kit (Biobox Biotech, Nanjing, China), and Caspase-12 Fluorometric Assay Kit (BioVision, Mountain View, CA, USA) according to the manufacturers’ instructions.

### 4.7. Mitochondrial Membrane Potential Analysis

Mitochondrial membrane potential (MMP) was analyzed by flow cytometric analysis using Rhodamine 123 (Rh123; Beyotime Biotechnology, Shanghai, China). After cells were exposed to Aβ25–35 with or without ginsenoside Re co-treatment, Rh123 was added to the media (2 μM final concentration) at 37 °C for 30 min in the dark. After incubation, cells were detached using trypsin, centrifuged at 125× *g* for 5 min, and resuspended in PBS. Amnis flow cytometry was used for detection.

### 4.8. Measurement of Cellular ATP Content

Levels of ATP in cells were determined with an ATP assay kit (Beyotime Biotechnology, Shanghai, China). Briefly, after washing with PBS, cells were lysed and centrifuged. Subsequently, 100 μL of ATP detection working solution was added to the supernatant, and the chemiluminescence of samples was measured according to the manufacturer’s instructions.

### 4.9. Intracellular ROS Generation Detection

Intracellular ROS was monitored using the fluorescent probe 2,7-dichlorofluorescein diacetate (DCFH-DA; Invitrogen, Carlsbad, CA, USA) as described previously [52].

### 4.10. Oxidative Stress Assays

Oxidative stress was assessed by measuring glutathione (GSH) levels and the activities of superoxide dismutase (SOD) and glutathione peroxidase (GPx). Supernatant protein concentrations were measured using a BSA kit (Beyotime Biotechnology). Levels of GSH and activities of SOD and GPx were measured using appropriate kits purchased from Beyotime Biotechnology according to the manufacturer’s instructions.

### 4.11. Reverse-Transcription and Real-Time PCR

Total RNA was extracted with TRIzol reagent (Invitrogen) according to the manufacturer’s instructions. Reverse transcription was performed using a PrimeScript RT reagent kit (Takara Biotechnology, Dalian, China). Real-time PCR was performed in a CFX 96 Connect™ Optics Module (Bio-Rad Laboratories, Hercules, CA, USA) using SYBR Green PCR Master Mix (Takara Biotechnology). Aliquots of cDNA were used for PCR using primer sets specific to Nrf2, GCLC, HO-1 and NQO1, and GAPDH as a control. Primers were as follows: Nrf2: 5′-AGCGACGGAAAGAGTATGA-3′, 5′-TGGGAGTAGTTGGCAGAT-3′; GCLC: 5′-TTGTTATGGCTTTGAGTG-3′, 5′-TCTGAGTTTGGAGGAGGG-3′; HO-1: 5′-AATGGTTCAGGCAACAGGG-3′, 5′-CTCCAGCAGTATGAGCAAAGTA-3′; NQO1: 5′-TGGTTTGAGCGAGTGTTC-3′, 5′-TATTCTCCAGGCGTTTCT-3′; GAPDH: 5′-GGTGAAGGTCGGAGTCAACGGA-3′, 5′-GAGGGATCTCGCTCCTGGAAGA-3′. Data were analyzed according to the 2^−ΔΔct^ method, as previously described [53].

### 4.12. Protein Extraction and Western Blotting

After treatments, cells were lysed with ice-cold RIPA lysis buffer (Beyotime Biotechnology) containing protease inhibitors. Total protein concentration was determined using a BSA kit. For detection of cyt c, mitochondrial, and cytosolic fractions were prepared using a Mitochondria/Cytosol Fractionation kit (Abcam, Cambridge, UK). For detection of Nrf2 nuclear transfer, a NE-PER™ Nuclear and Cytoplasmic Extraction Reagents kit (Thermo Scientific, Waltham, MA, USA) was used. Western blotting was conducted as previously described [54]. Antibodies against Bcl-2, Bax, cytochrome c, Nrf2, p-JNK, JNK, p-ASK-1, ASK-1, GAPDH, Lamin B, and COX IV were supplied by Cell Signaling Technology (Danvers, MA, USA) or Abcam. Secondary antibodies were obtained from Cell Signaling Technology or Proteintech (Chicago, IL, USA).

### 4.13. Statistical Analysis

Statistical analysis was performed using Prism 6.0 software (GraphPad Software, San Diego, CA, USA). Data are expressed as mean ± standard deviation (SD) from at least three independent experiments. Statistical analysis was performed using a one-way analysis of variance (ANOVA) test followed by a Tukey post-hoc test, * *p* < 0.05, ** *p* < 0.01, *** *p* < 0.001, **** *p* < 0.0001 versus control; ^#^ *p* < 0.05, ^##^ *p* < 0.01, ^###^ *p* < 0.001, ^####^ *p* < 0.0001 versus Aβ treatment alone; ^&^ *p* < 0.05, ^&&^ *p* < 0.01 versus the cells treated with Aβ and 25 uM Ginsenoside Re.
